# Noun and verb processing in aphasia: Behavioural profiles and neural correlates

**DOI:** 10.1016/j.nicl.2018.01.023

**Published:** 2018-01-31

**Authors:** Reem S.W. Alyahya, Ajay D. Halai, Paul Conroy, Matthew A. Lambon Ralph

**Affiliations:** a Neuroscience and Aphasia Research Unit, Division of Neuroscience & Experimental Psychology, Manchester Academic Health Science Centre, University of Manchester, United Kingdom; b King Fahad Medical City, Riyadh, Saudi Arabia

**Keywords:** Noun and verb processing, Aphasia, Lesion-symptom mapping, Principal component analysis, Imageability

## Abstract

The behavioural and neural processes underpinning different word classes, particularly nouns and verbs, have been a long-standing area of interest in psycholinguistic, neuropsychology and aphasiology research. This topic has theoretical implications concerning the organisation of the language system, as well as clinical consequences related to the management of patients with language deficits. Research findings, however, have diverged widely, which might, in part, reflect methodological differences, particularly related to controlling the psycholinguistic variations between nouns and verbs. The first aim of this study, therefore, was to develop a set of neuropsychological tests that assessed single-word production and comprehension with a matched set of nouns and verbs. Secondly, the behavioural profiles and neural correlates of noun and verb processing were explored, based on these novel tests, in a relatively large cohort of 48 patients with chronic post-stroke aphasia. A data-driven approach, principal component analysis (PCA), was also used to determine how noun and verb production and comprehension were related to the patients' underlying fundamental language domains. The results revealed no performance differences between noun and verb production and comprehension once matched on multiple psycholinguistic features including, most critically, imageability. Interestingly, the noun-verb differences found in previous studies were replicated in this study once un-matched materials were used. Lesion-symptom mapping revealed overlapping neural correlates of noun and verb processing along left temporal and parietal regions. These findings support the view that the neural representation of noun and verb processing at single-word level are jointly-supported by distributed cortical regions. The PCA generated five fundamental language and cognitive components of aphasia: phonological production, phonological recognition, semantics, fluency, and executive function. Consistent with the behavioural analyses and lesion-symptom mapping results, both noun and verb processing loaded on common underlying language domains: phonological production and semantics. The neural correlates of these five principal components aligned with existing models of language and the regions implicated by other techniques such as functional neuroimaging and neuro-stimulation.

## Highlights

• Developed new matched set of nouns and verbs to assess naming and comprehension.
• No noun-verb processing differences once matched on multiple psycholinguistic features.
• Neural correlates of noun and verb processing jointly supported within the cortex.
• Stroke aphasia is modulated by independent but interactive language domains.
• Core language functions are supported by grey and white matter regions.

## 1 Introduction

### 1.1 Behavioural status of noun and verb processing in aphasia

The assessment and treatment of individuals with aphasia secondary to acquired brain injury, such as stroke, provide us with a window into the behavioural and neural systems underpinning language. Several aphasiological studies have investigated the effect of word class (particularly nouns and verbs) in individuals with aphasia. Typically, the aphasia clinical profile involves greater difficulties with verb processing (compared to nouns), both during comprehension and production. This verb processing deficits potentially undermining lexical retrieval, sentence comprehension and production, and ultimately connected speech production and the engagement in conversations. Several competing linguistic explanations have been proffered to account for word class effects in aphasia. The lexical account claims that nouns and verbs are stored separately in the mental lexicon and the noun-verb dissociation results from selective damage to accessing either the noun or the verb lexicon at the lexical stage of word production (Hillis and Caramazza, 1995; Miceli et al., 1988; Miceli et al., 1984). The semantic account proposes that verbs are more difficult because they are semantically more complex. Verbs tend to be lower in imageability (the degree to which a word can generate a mental image and/or sensory experience) than nouns, and have less perceptual features (Bird et al., 2000; Breedin et al., 1998; McCarthy and Warrington, 1985). The syntactic account suggests that greater verb deficits are a consequence of the syntactic complexity of verbs given their syntactic role in sentences (Kim and Thompson, 2000; Thompson, 2003). Some researchers argue that it is difficult for this account to explain noun and verb dissociation observed during single-word production (Berndt et al., 1997a), although the proponents of the syntactic account would argue that syntactic structures associated with verbs are engages even when the verb was produced in isolation (Kim and Thompson, 2000). Lastly, the morphological account suggests that verbs are more difficult to process because they are morphologically more complex, as they carry a greater number of inflectional morphemes in most languages (Badecker and Caramazza, 1991; Tsapkini et al., 2002). Though this account is challenged by studies revealing noun-verb dissociation in languages with no morphological differences between nouns and verbs, such as Chinese (e.g., Bates et al., 1991).

The pattern of results and the associated theories concerning the noun-verb literature in aphasia have been inconsistent. Some researchers have emphasised a noun-verb double dissociation (e.g., Miceli et al., 1988; Miceli et al., 1984), whereas more recent studies have shown greater verb deficits compared to nouns (e.g., Luzzatti et al., 2002; Mätzig et al., 2009). Differential noun-verb processing has also been compared with aphasia classifications, proposing a potential association between fluent aphasia with noun deficits, and non-fluent aphasia with verb deficits (e.g., Bates et al., 1991; Hillis and Caramazza, 1995; Laiacona and Caramazza, 2004; Zingeser and Berndt, 1988). This view has been challenged, however, by studies showing greater verb deficits compared to nouns among: (i) individuals with fluent aphasia (e.g., Berndt and Haendiges, 2000; Jonkers and Bastiaanse, 1998), and (ii) individuals from both fluent and non-fluent aphasia groups (e.g., Bastiaanse and Jonkers, 1998; Berndt et al., 2002; Jonkers and Bastiaanse, 1996; Luzzatti et al., 2002; Mätzig et al., 2009). An extensive theoretical review by Vigliocco et al. (2011) demonstrated that all reports on patients with large noun-verb dissociation in the literature up to 2011 could be accounted for by three main factors: (i) task, whether it tackles lexical retrieval or sentence processing and phrasal construction; (ii) cross-linguistic differences between the use of nouns and verbs in sentences, in term of morphological markers and syntactic complexity; and (iii) semantic distinctions between nouns and verbs. The importance of semantic differences between nouns and verbs has also be emphasised in a recent review, which notes that cross-linguistically nouns refer to objects and verbs usually predict actions and events (Kemmerer, 2014).

The focus of the current study was lexical processing and the semantic distinction between nouns and verbs rather than sentence processing and the morpho-syntactic disparities, and this was addressed using single-word tasks. There are three potential reasons for the inconsistent findings concerning the noun-verb differences at single-word level in the literature, which were tackled in the present study by developing a new set of matched materials to assess both production and comprehension of nouns and verbs. The first issue identified in the literature is variation of psycholinguistic features between noun and verb items utilised in different studies and the challenge of adequate control over these variables. In early studies, noun and verb items were not matched on any psycholinguistic variables (e.g., Bates et al., 1991; Hillis and Caramazza, 1995; Miceli et al., 1984). Other studies matched the noun and verb items on word frequency (e.g., Bastiaanse and Jonkers, 1998; Berndt et al., 2002; Berndt and Haendiges, 2000; Berndt et al., 1997a; Berndt et al., 1997b; Jonkers and Bastiaanse, 1996, Jonkers and Bastiaanse, 1998; Laiacona and Caramazza, 2004), age-of-acquisition (Druks and Carroll, 2005; Mätzig et al., 2009), frequency and length (e.g., Miceli et al., 1988; Zingeser and Berndt, 1988), age-of-acquisition, frequency and familiarity (e.g., Luzzatti et al., 2002), and frequency, familiarity, length and visual complexity (e.g., Shapiro and Caramazza, 2003). These studies failed to control for other variables, in particular, word imageability, which often has a strong effect on performance in aphasia, and it has been suggested that the relative verb deficits ceases to exist once imageability was controlled for (Bird et al., 2000). A second potential issue in the literature is that the vast majority of studies focused on production. Only few studies have investigated comprehension (e.g., Berndt et al., 1997b; Miceli et al., 1988). Finally, the third potential factor relates to the fact that the majority of the studies in the literature are single case (e.g., Druks and Carroll, 2005; Hillis and Caramazza, 1995; Shapiro and Caramazza, 2003; Zingeser and Berndt, 1988) or case-series studies (e.g., Bastiaanse and Jonkers, 1998; Berndt et al., 2002; Bird et al., 2000; Miceli et al., 1988; Miceli et al., 1984), with only few group studies (Jonkers and Bastiaanse, 1996; Luzzatti et al., 2002). With small samples it is possible, of course, to end up with divergent data, and it is much harder to relate performance on nouns and verbs to the variation of aphasiological presentation not only in terms of aphasia classification but to more specific components of aphasia (e.g., phonological abilities, semantics, fluency and so on).

In the current study, these methodological challenges were addressed by investigating noun and verb processing using a noun-verb set matched on multiple psycholinguistic variables simultaneously including word imageability, frequency, familiarity, age-of-acquisition, length and visual complexity. A set of matched materials was developed to assess both production and comprehension on a large cohort of patients with chronic post-stroke aphasia, including a wide range of aphasia classifications beyond Broca's and anomic aphasia.

### 1.2 Neural correlates of noun and verb processing

Noun-verb differences have also been linked to the neuroanatomical bases of noun and verb processing. One view posits an, at least, partially segregated representation of noun and verb processing, with verb processing mainly supported by the left frontal cortex (left inferior and superior frontal gyri and pre-frontal cortex), and noun processing largely supported by left temporal regions (primarily middle fusiform gyrus, anterior and lateral temporal regions). These effects have been shown in both production and comprehension, and evidence for this view comes from neuropsychological (e.g., Damasio and Tranel, 1993; Daniele et al., 1994), functional neuroimaging (e.g., Shapiro et al., 2006; Shapiro et al., 2005), cortical stimulation mapping studies (e.g., Lubrano et al., 2014) and repetitive transcranial magnetic stimulation (Cappelletti et al., 2008). The noun/verb stimuli in these studies, however, were matched on frequency and length, or were not matched on any psycholinguistic variables. In contrast to this view, there is evidence that wide cortical regions jointly correlate with noun and verb processing, including the left frontal, parietal and temporal lobes. This view is supported by neuropsychological studies showing that verb deficits can result from lesions outside the left frontal lobe including left posterior temporal regions, parietal lobe, posterior lateral temporal-occipital junction, basal ganglia, insula, and/or extensive lesions involving fronto-temporal perisylvian area (e.g., Aggujaro et al., 2006; Kemmerer et al., 2012; Luzzatti et al., 2006; Tranel et al., 2001). Several functional neuroimaging studies also suggested common but distributed neural correlates of noun and verb processing, with activation observed in wide, overlapping set of brain regions within left frontal, temporal and parietal regions (e.g., Li et al., 2004; Siri et al., 2008). A review of the neural correlates of noun and verb processing in functional neuroimaging studies showed that the majority of regions that were selectively activated for one word class in some studies were found to be selectively activated for the other word class in different studies (Crepaldi et al., 2011). The authors argued that these inconsistencies suggest that the neural correlates of noun and verb processing are not segregated. A subsequent meta-analysis on functional neuroimaging studies suggested a distributed network correlating with noun and verb processing, including frontal, temporal and parietal regions (Crepaldi et al., 2013).

Some caution is needed when interpreting some of the earlier neuropsychological results, as most of them did not utilise accurate brain mapping techniques. More recently, with the use of MRI, sophisticated methods have been developed where lesion sites can be mapped precisely and then correlated with behavioural deficits. This methodology was used in the current study, while controlling for several psycholinguistic variables between the noun and verb stimuli.

### 1.3 Current study

In the current study, a well-matched set of noun and verb materials was created, which probed both single-word production and comprehension. To ensure clinically relevant findings, the word class effect was explored in a large and diverse, non-selected cohort of patients with chronic post-stroke aphasia. Since noun and verb processing has not been explored in relation to the wider context of the neuropsychological profile of patients with post-stroke aphasia, a data driven approach, principal component analysis (PCA), was used to explore the relationship between noun/verb processing and the underlying fundamental language domains that were extracted from a large neuropsychological assessment battery. This data driven approach accounts for the graded multidimensional nature of post-stroke aphasia (Butler et al., 2014), and has been showed to be robust and replicable in previous studies, even when increasing the sample size or adding/changing the behavioural measures (Butler et al., 2014; Halai et al., 2017; Lacey et al., 2017; Mirman et al., 2015). Additionally, this approach can build group level models (the overall output of the PCA structure and components) and also provide useful subject specific detail (the graded nature of the reconstructed factor scores). This approach helps to reveal a picture of the various underlying variations found in a heterogeneous clinical sample, which is typical in post-stroke aphasia. In addition, by applying a varimax rotation, orthogonal components are retained but it tends to become simpler to interpret the relationship between the patients' language abilities and the extracted components. For these reasons, PCA was utilised in this study to explore how performance on tasks related to noun/verb processing are related to fundamental language components and to what degree. Exploration of the neural correlates of noun and verb processing, as well as the underpinning principal language components, was achieved using lesion-symptom mapping.

## 2 Methods

### 2.1 Participants

Forty-eight stroke patients (single left ischaemic or haemorrhagic stroke) with chronic aphasia participated in this study (34 males and 14 females). The Boston Diagnostic Aphasia Examination (BDAE: Goodglass and Kaplan, 1983) was administered to each participant, and their aphasia was classified using the BDAE standard aphasia classification criteria. Their age ranged between 44 and 87 (Mean = 63.31, SD = 11.8) and educational level varied from 9 to 19 years (Mean = 12.58, SD = 2.5). Participants were recruited from the North West of England via stroke community support groups and speech and language therapy services. They were recruited on the basis that they had a single left hemispheric stroke and were at least 12 months post-stroke. All participants were native English speakers with normal or corrected-to-normal hearing and/or vision. Participants were not selected based on their neuropsychological profile or lesion location, and no restrictions were placed according to aphasia severity or classification, in order to sample the full range of severities and classifications of aphasia. The exclusion criteria included any contraindications for MRI scanning, being pre-morbidly left-handed and having more than one stroke or any other significant neurological conditions. Demographic details on all participated are available in Table 1. Informed consent was obtained from all participants prior to participation under approval from the local ethics committee.

**Table 1:** Participants demographic information arranged according to their lesion volume.

**Participant**	**Age** (years)	**Gender**	**Education** (years)	**Time-post onset** (months)	**Lesion volume** (voxels 2 mm^3^)	**BDAE aphasia classification**	**BDAE aphasia severity**^a^
NH	44	Female	16	21	175	Anomia	5
Ebe	54	Female	11	65	1526	Anomia	4.5
KA	70	Male	11	40	3311	Anomia	4.5
PBL	47	Female	16	34	3897	Conduction	2
DL	49	Male	19	37	4538	Anomia	4
TK	69	Male	11	49	4773	Conduction	3
MH	67	Male	11	17	4879	Conduction	3
DCs	49	Female	13	62	5273	Anomia	4
KS	64	Male	12	71	5822	Transcortical Sensory	3.5
RH	66	Male	17	16	6557	Conduction	3
WE	65	Male	10	85	6607	Anomia	3.5
GHa	56	Male	16	18	6974	Anomia	5
DF	46	Female	11	81	6975	Anomia	3.5
RL	85	Male	9	48	7854	Anomia	4
JBr	69	Female	19	37	8118	Anomia	3.5
EBo	46	Male	11	62	8437	Anomia	3.5
WC	87	Male	9	21	8528	Anomia	3.5
BH	70	Male	11	77	8788	Anomia	3.5
JS	71	Female	19	56	9159	Anomia	3.5
AL	54	Female	12	129	9767	Anomia	4.5
MB	45	Male	14	27	10,409	Anomia	4
DM	53	Male	17	100	11,915	Broca's	3.5
PW	75	Female	11	168	12,057	Mixed non-fluent	2
JW	83	Male	9	24	12,131	Broca's	2
MAd	58	Female	15	280	12,699	Anomia	3
RR	60	Male	13	58	13,080	Broca's	2
AD	77	Female	11	74	13,577	Broca's	3
KL	60	Male	13	76	14,625	Mixed non-fluent	1
GP	60	Male	11	49	16,433	Anomia	4
JSc	82	Male	12	122	18,163	Broca's	2
AG	59	Male	15	184	18,392	Broca's	3.5
DC	55	Male	13	56	18,632	Broca's	2.5
CH	44	Female	11	32	18,948	Anomia	4
AS	74	Male	11	24	19,500	Global	1
MD	74	Male	11	42	22,732	Mixed non-fluent	1
AB	52	Male	13	90	22,948	Anomia	3.5
PR	73	Female	12	54	23,863	Transcortical Mixed	3
GL	52	Male	12	73	26,218	Broca's	2.5
GD	68	Male	11	53	31,317	Mixed non-fluent	1
DB	65	Male	12	102	31,599	Mixed non-fluent	1
DR	64	Male	11	36	33,239	Mixed non-fluent	2
JM	58	Male	13	74	33,239	Global	1
Gho	80	Male	11	64	33,678	Mixed non-fluent	2
JBo	79	Male	13	56	34,242	Mixed non-fluent	1
PM	74	Male	11	117	36,877	Broca's	3
SL	63	Male	11	76	37,822	Global	1
CF	54	Female	11	118	40,313	Mixed non-fluent	1
DBb	70	Male	12	68	41,379	Global	1

BDAE = Boston Diagnostic Aphasia Examination.

a As measured by the BDAE aphasia severity rating: on a 5-point scale (1 indicates severe aphasia).

### 2.2 Materials

A noun and verb set was created, which included 32 items in each word class matched pairwise on word imageability, frequency, familiarity, age-of-acquisition, length and visual complexity of the pictorial image. All noun and verb items were drawn from the Object and Action Naming Battery (OANB: Druks and Masterson, 2000), and the psycholinguistic values for each noun and verb item were drawn from published norms, the rating scales and the statistical information on the psycholinguistic features of the matched stimulus sets are provided in Table 2. A computerised algorithm was used to create the matched set, in order to balance between matching the nouns and verbs for a wide range of psycholinguistic variables and, at the same time, to maximise the number of items in each word class. This matched set was used to examine noun-verb naming differences. Each picture was centrally presented for 10 s on a laptop screen, and participants were instructed to name it aloud using a single word. They were asked either to name the object, if it was an object picture, or to say what is happening in the picture or what is the person in the picture doing, if it was an action picture. No further cues were provided. Responses were recorded continuously with audio recording for offline coding for accuracy and naming errors. The initial response was entered into the accuracy analysis and was deemed to be correct by the examiner if the response corresponded to the name of the item as indicted by the OANB. Multiword responses that contained the target were deemed correct, if the initial noun (for object pictures) or verb (for action pictures) was the target response (e.g., ‘witch on a broom’ for ‘witch’; and ‘opening the door’ for ‘opening’).

**Table 2:** Mean (and SD) of psycholinguistic variable of the noun and verb sets, and results from independent *t*-tests that were carried out to examine the differences between these mean values.

**Psycholinguistic variable**	**Noun set**	**Verb set**	***t*-test**
Imageability^a^	5.03 (0.58)	4.8 (0.45)	*p* = 0.09
Age-of-acquisition^a^	2.88 (0.69)	2.65 (0.72)	*p* = 0.19
Familiarity^a^	3.52 (1.29)	4.02 (1.53)	*p* = 0.17
Lemma frequency^b^	5359.25 (7388.47)	6887.53 (9949.5)	*p* = 0.5
Log frequency^b^	3.4 (0.56)	3.46 (0.6)	*p* = 0.73
Word length^c^	3.84 (0.79)	3.53 (0.61)	*p* = 0.09
Visual complixity^a^	3.7 (1.33)	3.5 (1.48)	*p* = 0.59

a Ratings on a 7-piont scale (Masterson and Druks, 1998).

b British National Corpus (British National Corpus Consortium, 2007).

c Number of phonemes of the nouns and the verb stems.

A comprehension test was also developed using the same items. This picture-to-word matching test involved the target picture above five written-word choices. The choices included the target (e.g., ‘brush’) and four distractors from the same word class, two were semantically related to the target (e.g., ‘comb’ and ‘hair’) and two were unrelated (e.g., ‘sword’ and ‘crack’). All verbs were presented in the present continuous tense to avoid confusion with nouns (e.g., ‘fishing’). This picture-to-word matching test was used to examine noun-verb comprehension differences. The items were presented in a randomised order on a laptop screen using E-prime® version 1.2 (Psychology Software Tools Inc., Sharpsburg, Philadelphia) with simultaneous auditory and visual presentation of each word. This task was not timed, and auditory repetition of the choices was provided if required.

The naming and picture-to-word matching tests were administered following an example item, and three practice items that were not included in the main test, to ensure that participants understood the task. During the practice trails, participants were trained to name the objects or the action with a single noun or verb, respectively. The naming tests were administrated first followed by the picture-to-word matching tests, in order to avoid any cueing effects on the naming tests. After the administration of each test, a break was taken followed by an administration of another test (not included in this study). In addition, in order to replicate previous studies that found noun-verb differences using a less matched items, a subset of 18 verbs and 18 nouns unmatched on their imageability, familiarity, frequency, age-of-acquisition and length (*p* = 0.05 to *p* < 0.0001) were used to examine differences between noun and verb production and comprehension.

The newly developed picture-to-word matching tests were piloted among eight English speaking healthy younger adults. The results showed 100% accuracy. However, six semantic distractors were identified by at least 25% of the participants as potential correct responses (e.g., ‘head’ for ‘brain’, ‘work’ for ‘office’). Therefore, they were replaced with different semantic distractors, and subsequently the test was piloted again with 100% accuracy and no issues with the distractors. A list of the noun and verb matched set used in the naming tests and the newly developed noun and verb picture-to-word matching tests are provided in the Supplementary Appendix A.

Normative data for the newly developed picture-to-word matching tests and name agreement for the noun and verb matched naming set were collected from twenty-five healthy elderly control participants (9 males and 16 females). Participants were native English-speakers, right handed, their age ranged between 61 and 86 (Mean = 71.64, SD = 5.37), and their educational level varied from 10 to 19 years (Mean = 14.4, SD = 3.1). All participants reported no history of any neurological condition or brain injury, and their scores on the Mini Mental State Examination (Folstein et al., 1975) were above 26 (Mean = 28.92, SD = 1.07). The results revealed high accuracy obtained by the groups as whole: 99.62% (range = 31–32) on the verb picture-to-word matching test; 99.5% (range = 31–32) on the noun picture-to-word matching test; 99.75% (range = 31–32) on the verb naming test; and 97.8% (range = 29–32) on the noun naming test. Pearson product-moment correlation revealed no correlation between accuracy and age (r = 0.097, *p* = 0.65) or education (r = 0.066, *p* = 0.75). These results indicated that neither age nor education level had an effect on accuracy performance.

### 2.3 Background neuropsychological assessments

An extensive neuropsychological battery that assesses language and cognitive abilities was utilised (described in Butler et al., 2014). This included: (1) subtests from the Psycholinguistic Assessments of Language Processing in Aphasia (PALPA: Kay et al., 1992): auditory discrimination using non-word (PALPA 1) and word minimal pairs (PALPA 2), and immediate and delayed repetition of non-words (PALPA 8) and words (PALPA 9); (2) tests from the Cambridge Semantic Battery (Bozeat et al., 2000): spoken word-to-picture matching, a written word-to-picture matching version of the same task, the picture version of the Camel and Cactus test, and the picture naming test; (3) the Boston Naming Test (Kaplan et al., 1983); (4) the 96-trial synonym judgment test (Jefferies et al., 2009); (5) the verb synonym judgment test (Alyahya, Halai, Conroy, & Lambon Ralph, 2018); (6) the spoken sentence comprehension task from the Comprehensive Aphasia Test (CAT: Swinburn et al., 2005); and (7) cognitive tests including: the Brixton Spatial Rule Anticipation Task (Burgess and Shallice, 1997), forward and backward digit span (Wechsler, 1987), and Raven's Coloured Progressive Matrices (Raven, 1962). Additional fluency measures (number of tokens, token-type ratio, mean length of utterance, and words-per-minute) were included, which were extracted after transcribing and coding responses from the ‘Cookie theft’ picture description task (BDAE: Goodglass and Kaplan, 1983) (described in Halai et al., 2017). Results of background neuropsychological assessments are available in Supplementary Appendix B.

Participants' scores on the newly developed noun/verb naming and picture-to-word matching tests as well as all background neuropsychological measures were converted into percentages and entered into a PCA. All input measures are automatically standardised using the default parameters of factor analysis in SPPS. This method then extracts orthogonal components that best explain the variance within the dataset. Factors with an eigenvalue >1.0 were extracted and varimax rotated. This orthogonal rotation allows for better interpretation of the underlying language and cognitive process as it maximises the loading of a single task to one component. The adequacy of the sample size for this PCA was determined using Kaiser-Meyer-Olkin measure of sampling adequacy and Bartlett's test of sphericity.

### 2.4 Acquisition and processing of neuroimaging data

High-resolution structural T1-weighted MRI scans were acquired on a 3T Philips Achieva scanner (Philips Healthcare, Best, The Netherlands) using an eight-element SENSE head coil. A T1-weighted inversion recovery sequence with 3D acquisition was employed, with the following parameters: repetition time = 9.0 ms, echo time = 3.93 ms, slice thickness = 1 mm, flip angle = 8, 150 contiguous slices, acquired voxel size = 1.0 × 1.0 × 1.0 mm^3^, matrix size = 256 × 256, field of view = 256 mm, inversion time = 1150 ms, SENSE acceleration factor 2.5, total scan acquisition time = 575 s.

**Fig. 1:**
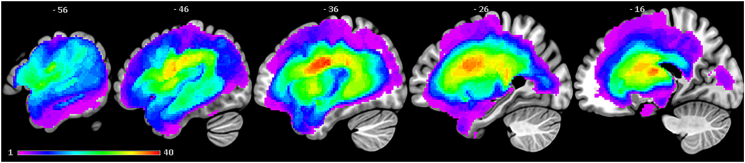
Lesion overlap map across 48 patients with post-stroke aphasia showing the distribution of patients' lesions. Colour scale indicates number of patients with a lesion in that voxel (threshold = 1–40). The maximum number of patients who had a lesion in one voxel was 40 (MNI coordinate: −38, −9, 24; central opercular cortex).

Patients' structural MRI scans were pre-processed with Statistical Parametric Mapping software (SPM8: Wellcome Trust Centre for Neuroimaging, http://www.fil.ion.ucl.ac.uk/spm/) running under Matlab 2012a. The first step was to strip non-brain tissue from the T1 images using an optimised brain extraction tool (OptiBET: Lutkenhoff et al., 2014). The resultant images were then normalised into standard Montreal Neurological Institute (MNI) space using a modified unified segmentation-normalisation procedure optimised for focal lesioned brains (Seghier et al., 2008). Structural imaging data from a healthy age and education matched control group (18 male and 4 female; mean age = 69.13 years, SD = 5.85, range = 59–80; and mean education = 13 years, SD = 2.66, range = 10–18) were used as a reference to identify abnormal tissue in the stroke group using an automated lesion identification procedure (Seghier et al., 2008). Structural MRI scans from these 22 healthy controls and all 48 post-stroke aphasia patients were entered into the segmentation-normalisation procedure. This procedure combines segmentation, bias correction and spatial normalisation through the inversion of a single unified model, which combines tissue classes (of grey and white matter, cerebral spinal fluid (CSF), and an additional tissue class for abnormal voxels), intensity bias correction and non-linear warping into the same probabilistic models that are assumed to generate subject-specific images (details available in Ashburner and Friston, 2005). Finally, images were smoothed with an 8 mm full-width-half-maximum Gaussian kernel, in order to account for the global intra-subject shape differences, and were used in the lesion-symptom mapping analyses. Each patient's lesion was automatically identified using a fully automated method based on fuzzy clustering (Seghier et al., 2008). The default parameters were used aside from the lesion definition ‘U-threshold’, which was set to 0.5 rather than 0.3 to create a binary lesion image. This modification was made after comparing the results obtained from a sample of patients to what would be nominated as lesioned tissue by an expert neurologist. The images generated for each patient were visually inspected with respect to the original scan and were then used to generate a lesion overlap map (Fig. 1), which primarily covers the left hemisphere area supplied by the middle cerebral artery (MCA) (Phan et al., 2005). The Seghier et al. (2008) procedure was selected because it is an objective and efficient method for large samples of patients (Wilke et al., 2011), in comparison to a labour intensive hand-traced lesion mask. It should be emphasised, that the procedure essentially detects areas of neural abnormality in an unexpected tissue class, and thus, identifies missing grey and white matter as well as areas of augmented CSF space.

### 2.5 Analysis of neuroimaging data

To identify the neural correlates of noun and verb processing, the normalised-smoothed T1-weighted images with continuous signal intensity values in each vowel across the whole brain were correlated with patients' individual behavioural measures using Voxel-Based Correlational Methodology (VBCM: Tyler et al., 2005) conducted in SPM8 and running under Matlab 2012a. This technique is a variant of voxel-lesion symptom mapping (VLSM: Bates et al., 2003) but, instead of using a binary classification for brain tissue (intact versus lesioned), a continuous measure of signal intensity is used and correlated with the behavioural data. Several VBCM analyses were conducted. First, noun and verb scores were analysed separately to identify the neural correlates associated with performance on noun versus verb production and comprehension. Individual scores on these tests (noun naming, verb naming, noun picture-to-word matching, and verb picture-to-word matching) were entered into separate analyses. Secondly, noun and verb scores on naming and picture-to-word matching tests were summed for each patient, to generate an overall naming and picture-to-word matching scores. These two measures were analysed separately, to identify the neural correlates associated with naming versus comprehension. These results were thresholded at *p* = 0.0005 voxel-level, cluster-level corrected using family-wise error (FWE) *p* < 0.05. This stringent threshold was used, in order to increase the specificity of the large clusters measured with each condition when they were examined in a separate model. Thirdly, whole brain direct contrasts were conducted between: (i) noun versus verb naming; (ii) noun versus verb picture-to-word matching; and (iii) naming versus picture-to-word matching. These direct contrasts were performed in order to identify regions that are uniquely associated with performance on one test over-and-above the other. Finally, patients' factor scores obtained from the PCA (of the entire aphasiological and neuropsychological assessments including the noun and verb measures) were entered simultaneously into a VBCM analysis. The identified clusters indicate where tissue concentrations uniquely correlate with a given factor over-and-above the other factors. This was done to identify the neural correlates of the extracted language and cognitive aphasia domains. As these factors are orthogonal, this analysis could either be done in one step with all factors entered simultaneously, or in several steps where each factor is entered in a separate model. The former method was followed to reduce the number of analyses, and it is believed that a single model that captures all (or as much) behaviours as possible is more elegant. The standard threshold of *p* = 0.001 voxel-level, cluster-level corrected using FWE *p* < 0.05 was used in the direct contrasts and the PCA-VBCM analyses, when several measures were entered simultaneously in the same model, as these effects are expected to be more subtle. The results of these VBCM analyses indicate which voxels' variation in tissue concentration corresponds to the variance in a given measure or factor score, while controlling for variation of other factors if included in the same model.

All VBCM analyses were carried out using multiple regression models on T1-weighted images, and test measures or PCA-factor scores entered as regressors of interest. Moreover, each patient's lesion volume (proxy of neurological severity) obtained from the output of the automated lesion identification procedure (Seghier et al., 2008) was entered as a covariate in subsequent VBCM analyses at the standard threshold of *p* < 0.001 voxel-level and cluster corrected using FWE *p* < 0.05. It is important to notice, however, that by partialling out lesion volume there is a high risk for type II error. Hence, all VBCM analyses were performed and reported in this paper once with the behaviours of interest only and once with a correction for lesion volume. This protocol was followed in order to account for both type I and type II errors. All anatomical results are described using labels based on the Harvard-Oxford atlas in MNI space (Desikan et al., 2006) and natbrainlab white matter connections atlas based on diffusion tensor tractography (Catani et al., 2012). All images were constructed using MRIcron (Rorden et al., 2007).

## 3 Results

### 3.1 Behavioural results

#### 3.1.1 Noun and verb processing in aphasia

##### 3.1.1.1 Accuracy

There was no significant difference between the noun and verb picture-to-word matching comprehension tests for the group as whole: noun test (Mean = 28.3, SD = 4.58, range = 15–32) and verb test (Mean = 28.7, SD = 3.78, range = 17–32). Furthermore, the performance of each participant was investigated, and only one patient (MD) showed a significant verb advantage (χ^2^(1) = 5.32, *p* = 0.021), while the remaining 47 patients showed no significant difference between noun and verb comprehension.

There was also no significant difference between the noun and verb naming tests for the group as whole: noun test (Mean = 17.5, SD = 10.7, range = 0–31) and verb test (Mean = 18.4, SD = 10.7, range = 0–32). Additionally, at a single-case level, only two patients out of 48 (RH and KS) showed a significant verb advantage in naming (χ^2^(1) = 8.25, *p* = 0.004, χ^2^(1) = 5.52, *p* = 0.019, respectively), while the remaining 46 patients had no significant noun-verb naming differences. Further information on the background neuropsychological assessments of patients MD, RH and KS is available in Supplementary Appendix B.

##### 3.1.1.2 Type of errors

To investigate any other behavioural differences between nouns and verbs, differences in the types of errors made between the two word classes during the picture-to-word matching comprehension tests were explored. The results showed no significant differences between the two word classes. For noun items: 88.2% of the errors were semantic errors and 11.8% were unrelated errors, and for verb items: 86.2% were semantic errors and 13.8% were unrelated errors.

The distribution of naming errors was also inspected. A total of 676 and 627 naming errors were elicited by the noun and verb pictures, respectively. Errors were classified according to a pre-specified error classification system and the percentages of errors for each category were calculated (results are illustrated in Table 3). Omissions constitute 58.7% naming errors elicited by noun pictures and 53.8% naming errors elicited by verb pictures. In further analysis, omissions were removed as they account for over half of the proportion of errors; rare errors (<10%: word class, preservations, initial phoneme and mixed errors) were collapsed into one category (others), and all four semantic error classes were collapsed to form one category (semantic errors). The results as illustrated in Table 3 revealed significant differences between two error classes. First, the proportion of semantic errors was greater for nouns compared to verbs. Second, partial name errors (name parts of the picture other than the target, either the object or an action other than the target verb) were more frequent for verbs compared to nouns.

**Table 3:** Error classification system and the distribution of errors (%) elicited by noun and verb picture stimuli.

**Error class**	**Verb example**	**Noun example**	**Verb pictures**	**Noun pictures**	**Following collapsing**⁎	
					**Verb pictures**	**Noun pictures**
Omission	–	–	53.8	58.7	–	–
Phonological	‘smiwing’ for ‘swimming’	‘stord’ for ‘sword’	6.70	6.20	14.48	15.05
Unrelated	‘putting’ for ‘shaving’	‘shoes’ for ‘comb’	3.98	6.36	8.62	15.41
Partial name	‘sitting’ for ‘reading’	‘wig’ for ‘judge’	10.35	3.80	22.41	9.32⁎⁎
Circumlocution	‘dripping blood’ for ‘bleeding’	‘underneath tree’ for ‘root’	7.16	3.25	15.52	7.89
Semantic-coordinate	‘throwing’ for ‘catching’	‘brush’ for ‘comb’	8.28	8.13	28.97	43.01⁎⁎
Semantic-superordinate	‘playing’ for ‘skating’	‘food’ for ‘fruit’	0.64	1.47		
Semantic-associative	‘winter’ for ‘skiing’	‘heavy’ for ‘weight’	4.45	7.25		
Semantic-subordinate	None	‘orange’ for ‘fruit’	0.00	0.88		
Word class	‘blood’ for ‘bleeding’	‘riding’ for ‘saddle’	3.02	1.62	10.00	9.32
Preservation	–	–	0.47	0.74		
Initial phoneme	‘s..’ for ‘skiing’	‘s…’ for ‘slide’	0.30	1.18		
Mixed	‘lauping’ for ‘smiling’	‘sair’ for ‘stool’	0.80	0.30		


⁎ Omissions removed from this analysis; and rare errors (word class, preservations, initial phoneme and mixed) were collapsed into one category (others), and the four semantic errors were collapsed to form one category (semantic errors).

⁎⁎ Significant differences between the proportion of errors elicited by noun versus verb stimuli (chi-square test, *p* < 0.05).

##### 3.1.1.3 Relation to aphasia classification

**Fig. 2:**
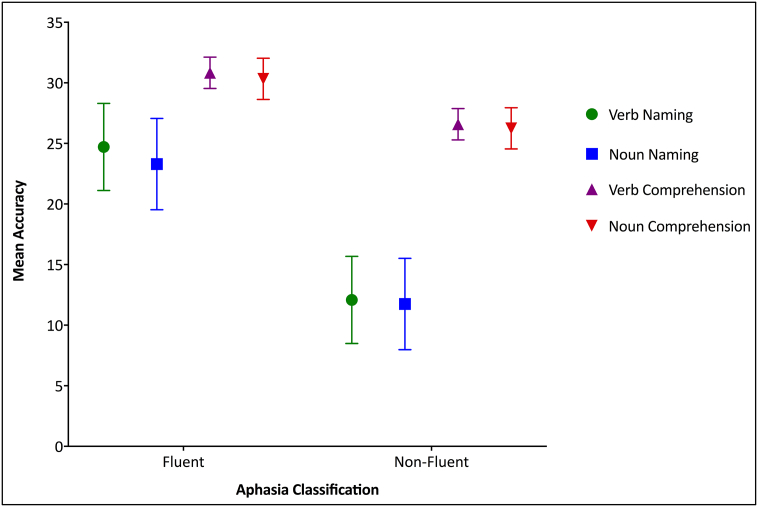
Participants' performance during noun and verb naming and comprehension tests: a main effect of aphasia group (advantage of fluent aphasia), a main effect of task (advantage of comprehension) and a lack of word class effect. Error bars represent 95% Confidence Interval.

A 2 × 2 × 2 mixed ANOVA was carried out with accuracy as the dependent variable and word class (nouns and verbs) and task (production and comprehension) as the within-subject conditions, and group (fluent and non-fluent aphasia) as the between-subject condition. Participants were classified as fluent or non-fluent according to their BDAE classification; in which participants with global, mixed non-fluent, Broca's or transcortical mixed aphasia were classified as non-fluent aphasia, and participants with anomic, conduction or transcortical sensory aphasia were classified as fluent aphasia. There were 25 participants in the fluent group and 23 in the non-fluent group. The results revealed a significant group effect (F(1,46) = 30.46, *p* < 0.0001, η2 = 0.39), significant effect of task (F(1,46) = 110.03, *p* < 0.0001, η2 = 0.7), and no significant effect of word class. There was a significant interaction between aphasia group and task (F(1,46) = 16.87, *p* < 0.0001, η2 = 0.26). This interaction was driven by higher naming scores among the fluent group (Mean = 48.2, SD = 13.24) compared to the non-fluent group (Mean = 22.57, SD = 20.34) (t(46) = 5.21, *p* < 0.0001); higher scores in response to picture-to-word matching test among the fluent group (Mean = 61.24, SD = 3.8) compared to the non-fluent group (Mean = 52.39, SD = 8.77) (t(46) = 4.6, *p* < 0.0001); higher picture-to-word matching scores (Mean = 61.24, SD = 3.78) compared to naming scores (Mean = 48.2, SD = 13.24) among the fluent group (t(24) = 5.26, *p* < 0.0001); and higher picture-to-word matching scores (Mean = 52.39, SD = 8.77) compared to naming scores (Mean = 22.57, SD = 20.34) among the non-fluent group (t(22) = 9.04, *p* < 0.0001). These results were corrected for multiple comparisons using Bonferroni correction. No further interactions were significant. These results (Fig. 2) confirmed the lack of word class effect and indicated an absence of association between noun and verb processing and aphasia groups (global, mixed non-fluent, Broca's or transcortical mixed versus anomic, conduction or transcortical sensory). This suggests that when using a matched set of noun-verb items, no single-word behavioural noun-verb differences emerge on either naming or comprehension. To confirm this, the performance of the group as whole was compared on a subset of 18 verb and 18 noun items unmatched on their imageability, familiarity, frequency, age-of-acquisition and length (*p* = 0.05 to *p* < 0.0001). Results showed a significant noun-verb naming differences in the advantage of nouns (Mean = 10.2, SD = 6.3) compared to verbs (Mean = 8.9, SD = 5.9) (t(47) = 5.26, *p* < 0.0001).

##### 3.1.1.4 Performance predictors

To explore the lack of word class effect further, word class along with other linguistic variables (imageability, frequency, age-of-acquisition, familiarity, world length and visual complexity) were entered into a multiple regression analysis to predict naming accuracy in post-stroke aphasia. The regression model was significant (R^2^ = 0.04, F(1,128) = 5.23, *p* = 0.024), with imageability appearing as the only significant predictor (B = 1.78, t = 2.29, *p* = 0.024). Another multiple regression was carried out to predict the accuracy on the picture-to-word matching test using the same predictors, and this model was also significant (R^2^ = 0.12, F(2,126) = 8.33, *p* < 0.0001), with imageability (B = 0.584, t = 2.46, *p* < 0.0001) and age-of-acquisition (B = 1.63, t = 3.06, *p* < 0.003) emerging as significant predictors. These results confirmed the lack of word class effect (once imageability and other factors are controlled) for the production and comprehension of single-words in post-stroke aphasia, as word class did not appear to be a significant predictor in both analyses. The results also suggest that imageability does account for performance in aphasia albeit not being the main/only predictor.

#### 3.1.2 Identifying principal language and cognitive components

The adequacy results revealed that the sample size was adequate for this PCA analysis (Kaiser-Meyer-Olkin = 0.79) and correlations between items were sufficiently large for this PCA (Bartlett's test = 1483.4, *p* < 0.0001). The PCA results produced five component factors, which accounted for 81.65% of variance (Factor 1: 51.45%, Factor 2: 11.5%, Factor 3: 7.8%, Factor 4: 6.27% and Factor 5: 4.6%). Following orthogonal rotation, the loadings of each measure on a particular factor (Table 4) allowed behavioural interpretation of fundamental language and cognitive domains. Measures that loaded heavily on the first factor were repetition and naming tests (e.g., word and non-word repetition, BNT and OANB), and thus this factor was interpreted as ‘phonological production’. Measures that loaded heavily on the second factor were either receptive or expressive tests that required processing of the meaning (e.g., synonym judgments, picture-to-word matching and OANB naming), and therefore this factor was interpreted as ‘semantics’. Measures that loaded heavily on the third factor were fluency measures (e.g., number of tokens and mean length of utterance), and hence this factor was interpreted as ‘fluency’. Measures that loaded heavily on the fourth factor were phonemic discrimination tests (word and non-word minimal pairs), and therefore this factor was interpreted as ‘phonological recognition’. Finally, measures that loaded heavily on the fifth factor were both executive function tests (Brixton spatial anticipation and Raven's progressive matrices), and thus this factor was interpreted as ‘executive functions’. The loadings of the two memory tests (forwards digits span, and backwards digit span) loaded on phonological production and fluency factors, respectively. This is expected, as both tests require intact production abilities for successful performance. Note that the noun and verb tests loaded together across similar factors, which indicates that successful performance for the two word classes is driven by common fundamental language domains: semantics and phonological production. In addition, the naming tests loaded heavily on both phonological production and semantic factors, indicating that naming performance require intact semantic and phonological production processes, and this is consistent with previous findings (Butler et al., 2014; Schwartz et al., 2006).

**Table 4:** Loadings of behavioural tests on factors extracted from rotated PCA.

**Test**	**Factor 1** **Phonological production**	**Factor 2** **Semantics**	**Factor 3** **Fluency**	**Factor 4** **Phonological recognition**	**Factor 5** **Executive functions**
Word repetition - immediate	**0.917**	0.175	0.094	−0.015	−0.070
Word repetition - delayed	**0.904**	0.234	0.138	0.166	−0.036
Non-word repetition - immediate	**0.896**	0.092	0.115	0.226	−0.152
Non-word repetition - delayed	**0.855**	0.070	0.203	0.313	−0.038
Cambridge 64-item naming	**0.786**	0.496	0.048	0.099	0.112
Boston Naming Test	**0.770**	0.449	0.066	0.087	0.155
Verb naming (OANB)	**0.748**	**0.536**	0.093	−0.070	0.164
Noun naming (OANB)	**0.736**	**0.617**	0.016	0.008	0.140
Forward digit span	**0.662**	0.153	0.225	0.279	−0.326
Camel and Cactus - pictures	0.166	**0.873**	0.172	0.075	0.107
Verb picture-to-word matching^a^	0.382	**0.829**	0.248	0.147	0.053
Noun picture-to-word matching^a^	0.211	**0.816**	0.220	0.100	0.108
Verb synonym judgment	0.338	**0.799**	0.249	0.115	0.003
96-Synonym judgment	0.374	**0.798**	0.291	0.150	−0.018
Written word-to-picture matching	0.117	**0.778**	0.002	0.449	0.050
Spoken word-to-picture matching	0.140	**0.756**	0.076	0.311	−0.350
Spoken Sentence Comprehension	0.496	**0.613**	0.234	0.210	−0.067
Raven's Coloured Progressive	0.219	**0.563**	0.027	0.204	**0.549**
Word-per-minute (fluency measure)	0.183	0.107	**0.837**	0.043	−0.101
Mean length of utterance (fluency measure)	0.239	0.347	**0.828**	0.023	0.078
Number of tokens (fluency measure)	−0.023	0.203	**0.811**	0.005	0.280
Backward digit span	0.495	0.183	**0.511**	0.399	−0.232
Non-word minimal pairs	0.199	0.222	−0.030	**0.893**	0.145
Word minimal pairs	0.219	0.320	0.125	**0.816**	0.005
Type-token ratio (fluency measure)	0.385	0.336	−0.077	0.029	−0.591
Brixton spatial anticipation test	0.058	0.446	0.214	0.213	**0.526**

Factor loadings > 0.5 are given in bold.

a Tests developed in this study.

### 3.2 Neuroimaging results

#### 3.2.1 Neural correlates of noun and verb processing

##### 3.2.1.1 Naming

**Fig. 3:**
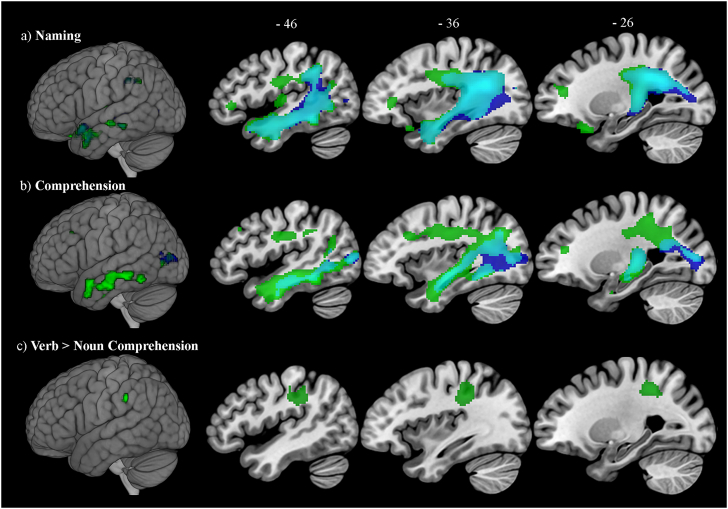
Lesion-symptom mapping showing overlapping (cyan) neural correlates of noun (blue) and verb (green) processing during (a) naming; and (b) comprehension. Results threshold *p* = 0.0005 voxel-level, cluster-level corrected using FWE *p* < 0.05. (c) Direct contrast showing neural correlated associated with verb over-and-above noun comprehension (green). Results threshold *p* = 0.001 voxel-level, cluster-level corrected using FWE *p* < 0.05.

The first set of VBCM analyses identified the neural correlates of noun and verb naming (Fig. 3a). The results showed overlapping regions for both noun and verb naming (Dice similarity coefficient = 0.77), this overlap extends from the left angular gyrus, posterior supramarginal gyrus, superior lateral occipital cortex through the posterior middle and inferior temporal gyri, temporal fusiform cortex and planum polare to the anterior middle temporal gyrus and temporal pole. The overlap also encompasses the posterior cingulate gyrus and the underlying white matter tracts corresponding to the anterior and posterior segments of the arcuate fasciculus, inferior longitudinal fasciculus and internal capsule. Additional frontal regions were identified for verb naming but not noun naming, this includes the left inferior frontal gyrus, medial frontal cortex, orbito-frontal cortex, frontal pole and pre-central gyrus. Significant clusters and peak MNI coordinates are listed in Table 5. To determine whether tissue variation in frontal regions were associated with performance on verb naming over-and-above noun naming, a further direct contrast was performed where both noun and verb naming scores were entering simultaneously in the model. The results showed that no regions were significantly related to verb naming over-and-above noun naming. Given that verb and noun naming were highly correlated (r = 0.94, *p* < 0.0001), also suggests that a common underlying language components support noun and verb processing.

**Table 5:** Neural correlates of significant clusters and peak MNI coordinates related to noun and verb naming and comprehension, and naming and comprehension (after summing noun and verb scores).

**Test**	**Location**	**Cluster size** (voxels)	**Z**	**MNI co-ordinates**		
				*x*	*y*	*z*
Verb naming	Angular gyrus Posterior inferior temporal gyrus Posterior segment of arcuate fasciculus Inferior longitudinal fasciculus Anterior middle temporal gyrus Posterior supramarginal gyrus Pre-central gyrus	12,546	5.25 5.14 4.80 4.49 4.39 4.32 4.11	−32 −46 −38 −40 −54 −46 −38	−54 −44 −44 −12 2 −46 −16	24 −16 16 −18 −24 38 30
	Posterior paracingulate gyrus Anterior cingulate gyrus Fontal medial cortex	253	4.30 4.22 3.49	4 −2 −10	52 44 50	−4 6 −8
	Frontal pole Temporal pole	432	4.41 3.48	−28 −40	42 1	16 −33
Noun naming	Angular gyrus Posterior segment of arcuate fasciculus Posterior inferior temporal gyrus Anterior middle temporal gyrus Inferior longitudinal fasciculus Anterior inferior temporal gyrus Posterior supramarginal gyrus Temporal pole	11,124	5.75 5.35 4.97 4.23 3.96 3.79 3.42 3.37	−32 −40 −44 −50 −40 −44 −40 −40	−54 −46 −42 −10 −2 −8 −52 5	24 14 −12 −22 −26 −34 34 −30
Verb comprehension	Posterior inferior temporal gyrus Inferior occipito-frontal fasciculus Planum polare Temporal occipital fusiform cortex Posterior middle temporal gyrus Anterior inferior temporal gyrus Precuneus Angular gyrus Post-central gyrus Medial frontal cortex Anterior supramarginal gyrus Pre-central gyrus Inferior longitudinal fasciculus Posterior segment of arcuate fasciculus	13,226	5.42 5.09 4.74 4.53 4.35 4.41 4.29 4.24 3.89 3.68 3.62 3.60 3.44 3.34	−46 −36 −50 −36 −66 −48 −10 −34 −24 −36 −38 −34 −33 −33	−44 −18 −60 −48 −36 −8 −46 −54 −40 20 −40 0 −72 −47	−14 −10 −6 −12 −12 −32 18 22 48 30 34 34 12 21
	Anterior cingulate gyrus Paracingulate gyrus Frontal pole Superior frontal gyrus	416	4.53 4.23 3.92 3.37	0 −6 −28 10	42 54 42 54	4 12 16 20
Noun comprehension	Superior lateral occipital cortex Inferior lateral occipital cortex Posterior middle temporal gyrus Precuneus Inferior longitudinal fasciculus Posterior inferior temporal gyrus	5056	4.90 4.42 4.22 4.12 4.06 3.85	−30 −46 −44 −12 −38 −44	−80 −80 −58 −46 −14 −42	8 6 −2 16 −16 −14
Verb comprehension > noun comprehension⁎	Post-central gyrus Anterior supramarginal gyrus Superior parietal lobule Precuneus	1347	3.96 3.50 3.44 3.42	−30 −62 −34 −12	−40 −34 −40 −50	44 36 40 44
Naming	Angular gyrus Posterior inferior temporal gyrus Cortico-spinal tract Inferior longitudinal fasciculus Posterior supramarginal gyrus Anterior middle temporal gyrus Posterior middle temporal gyrus Posterior supramarginal gyrus Posterior segment of arcuate fasciculus Temporal pole	12,446	5.61 4.98 4.74 4.6 4.41 4.34 4.15 4.61 4.16 3.20	−32 −46 −12 −40 −44 −50 −44 −56 −34 −34	−54 −44 −4 −12 −46 −10 −60 −42 −44 4	24 −14 −4 −18 36 −22 0 40 14 −38
	Frontal pole Orbito-frontal cortex	306	3.89 3.28	−28 −36	44 36	18 −14
Comprehension	Occipital fusiform gyrus Inferior occipito-frontal fasciculus Posterior inferior temporal gyrus Posterior middle temporal gyrus Angular gyrus Posterior temporal fusiform cortex Precuneus gyrus Inferior longitudinal fasciculus Inferior lateral occipital cortex Posterior segment of arcuate fasciculus	9333	4.94 4.77 4.77 4.59 4.38 4.22 4.21 4.04 3.49 3.28	−30 −36 −46 −52 −32 −38 −18 −34 −34 −34	−80 −16 −44 −62 −60 −38 −56 −8 −66 −48	8 −12 −14 −6 18 −16 26 −24 10 16
Naming > comprehension⁎	Angular gyrus Parietal operculum cortex Planum temporale Anterior middle temporal gyrus Posterior supramarginal gyrus Orbito-frontal cortex Frontal pole	11,607 441	5.14 4.72 4.57 4.32 4.14 3.85 4.26	−32 −32 −40 −54 −48 −26 −28	−54 −36 −44 2 −45 20 44	24 22 12 −26 38 −26 18

Results threshold *p* = 0.0005 voxel-level, cluster-level corrected using FWE *p* < 0.05.

⁎ Direct contrast at a lower threshold of *p* = 0.001 voxel-level, cluster-level corrected using FWE *p* < 0.05.

##### 3.2.1.2 Comprehension

Another set of VBCM analyses identified the neural correlates for noun and verb comprehension (Fig. 3b). The results showed overlapping regions (Dice similarity coefficient = 0.43), extending from the left angular gyrus, inferior lateral occipital cortex and temporal occipital fusiform cortex through the inferior and middle temporal gyri, and posterior temporal fusiform cortex. The overlap also encompasses the precuneus and the underlying white matter tracts corresponding to the inferior longitudinal fasciculus, posterior segment of the arcuate fasciculus, fornix and cortico-spinal tract. Additional frontal and parietal regions were identified for verb comprehension but not noun comprehension; this includes the left superior frontal gyrus, medial frontal cortex, frontal pole, pre-central gyrus, post-central gyrus, anterior supramarginal gyrus, and white matter tracts corresponding to the anterior segment of the arcuate fasciculus. The results from a whole brain direct contrast showed that left parietal regions encompassing the post-central gyrus and anterior supramarginal gyrus and white matter tracts corresponding to the anterior segment of the arcuate fasciculus were significantly related to verb comprehension over-and-above noun comprehension (Fig. 3c). Again, performance on the noun and verb comprehension tests was highly correlated (r = 0.8, *p* < 0.0001). Significant clusters and peak MNI coordinates are listed in Table 5.

#### 3.2.2 Neural correlates of production and comprehension

**Fig. 4:**
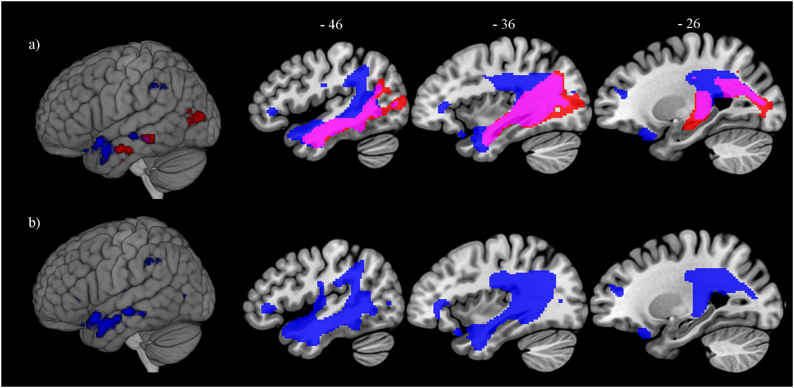
Lesion-symptom mapping showing (a) overlapping (pink) neural correlates of naming (blue) and comprehension (red). Results threshold *p* = 0.0005 voxel-level, cluster-level corrected using FWE *p* < 0.05; and (b) direct contrast showing the neural correlates of naming over-and-above comprehension (blue). Results threshold *p* = 0.001 voxel-level, cluster-level corrected using FWE *p* < 0.05.

Due to the shared temporal and parietal regions associated with naming and comprehension for both nouns and verbs, a further set of VBCM analyses were carried out to identify the neural correlate of production and comprehension irrespective of word class (Fig. 4a). The results showed overlapping regions for both production and comprehension (Dice similarity coefficient = 0.6), extending from the left angular gyrus and superior and inferior lateral occipital cortex, through the posterior inferior and middle temporal gyri to the anterior middle temporal gyrus. The overlap also encompasses the planum polare and the underlying white matter tracts corresponding to the posterior segment of the arcuate fasciculus, inferior longitudinal fasciculus, cortico-spinal tract, fornix, cortico-ponto-cerebellum tract, and internal capsule. Additional regions were associated with naming but not comprehension; this involves the left supramarginal gyrus, parietal operculum cortex, temporal pole, orbito-frontal cortex, inferior frontal gyrus and frontal pole, and the white matter tract corresponding to the anterior segment of the arcuate fasciculus. A whole brain direct contrast was performed to determine whether these additional tissue variations were associated with performance on naming over-and-above comprehension (Fig. 4b). The results showed that the left angular gyrus, posterior supramarginal gyrus, parietal operculum cortex, planum temporale, planum polare, anterior middle temporal gyrus, frontal pole, and orbito-frontal cortex and the white matter tracts corresponding to the anterior segment of the arcuate fasciculus were related to naming over-and-above comprehension, whereas no regions were associated with comprehension over-and-above naming. The high correlation between the two tasks (r = 0.73, *p* < 0.0001), could reflect the severity of aphasia that might affect performance on both tasks, or it might suggest common underlying language components that could be shared across production and comprehension. Significant clusters and peak MNI coordinates are listed in Table 5.

#### 3.2.3 Neural correlates of principal language and cognitive components

**Fig. 5:**
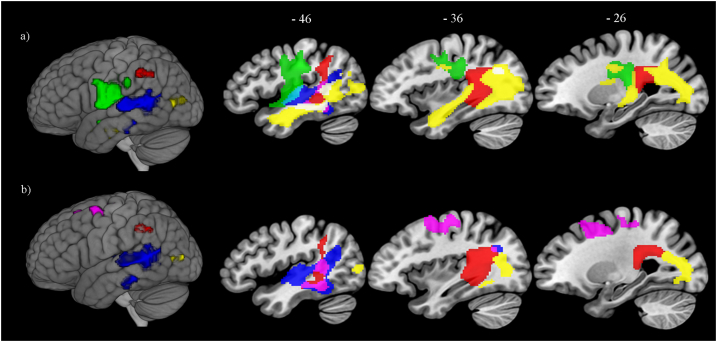
Lesion-symptom mapping on principal components extracted from PCA: phonological production (red), semantics (yellow), fluency (green), phonological recognition (blue), and executive functions (violet). (a) Results threshold *p* = 0.001 voxel-level, cluster-level corrected using FWE *p* < 0.05 (b) Results threshold *p* = 0.005, cluster-level corrected using FWE *p* < 0.05 while including lesion volume as a covariate.

Results from the VBCM analysis that identified the neural correlates of fundamental language and cognitive components are shown in Fig. 5a and significant clusters and peak MNI coordinates are listed in Table 6. Performance on the phonological production factor was uniquely associated with parietal operculum cortex. Performance on the phonological recognition factor was uniquely associated with the left posterior superior temporal gyrus, Heschl's gyrus (including H1 and H2), and posterior superior and inferior temporal gyri. Performance on the semantic factor was uniquely associated with left superior lateral occipital cortex, occipital fusiform gyrus, temporal occipital fusiform cortex, anterior inferior temporal gyrus, anterior temporal fusiform cortex, anterior middle temporal gyrus, temporal pole and precuneus. Performance on the fluency factor was uniquely associated with left frontal and partial regions involving the anterior supramarginal gyrus, central opercular cortex, pre- and post-central gyri, posterior parahippocampal gyrus, planum polare, parietal operculum cortex and the white matter tracts corresponding to frontal aslant tract, cortico-spinal tract, and internal capsule. There were no significant clusters identified for executive function factor. This factor, however, correlated with left frontal regions at a slightly lower threshold (*p* = 0.005 voxel-level, cluster corrected using FWE *p* < 0.05). This cluster included the left superior frontal gyrus, paracingulate gyrus, supplementary motor cortex and pre-central gyrus.

**Table 6:** Neural correlates of significant clusters and peak MNI coordinates related to language and cognitive factors extracted from PCA with and without accounting for lesion volume.

**Principal component factor**	**Location**	**Cluster size** (voxels)	**Z**	**MNI co-ordinate**		
				*x*	*y*	*z*
Factor 1: Phonological production	Posterior segment of arcuate fasciculus Angular gyrus Parietal operculum cortex Posterior supramarginal gyrus Inferior longitudinal fasciculus	3905	4.51 4.41 4.30 3.85 3.79	−40 −34 −32 −50 −34	−44 −54 −36 −46 −46	12 24 22 40 0
Factor 2: Semantics	Superior lateral occipital cortex Precuneus cortex Inferior longitudinal fasciculus Anterior temporal fusiform cortex Anterior inferior temporal gyrus Posterior inferior temporal gyrus Posterior middle temporal gyrus Anterior middle temporal gyrus Temporal pole	8326	5.53 5.01 4.67 4.67 4.28 4.12 4.04 3.90 3.78	−28 −18 −38 −38 −44 −46 −44 −48 −42	−74 −56 −8 −8 −10 −42 −58 −6 4	14 26 −20 −22 −32 −14 −2 −26 −26
Factor 3: Fluency	Central opercular cortex Post-central gyrus Pre-central gyrus Anterior segment of arcuate fasciculus	6008	4.78 4.48 3.58 3.30	−66 −66 −50 −37	−6 −14 −4 −19	8 16 40 25
Factor 4: Phonological recognition	Planum temporale Middle temporal gyrus Heschl's gyrus Angular gyrus Posterior supramarginal gyrus Posterior superior temporal gyrus	4088	4.42 4.31 3.96 3.40 3.36 3.32	−60 −54 −52 −46 −46 −46	−30 −44 −20 −52 −48 −38	8 6 6 20 14 6
Factor 5: Executive functions⁎	Superior frontal gyrus Paracingulate gyrus Supplementary motor areas pre-central gyrus	1085	3.65 3.36 3.27 2.96	−22 −6 −10 −12	20 30 −8 −16	52 32 52 48

Results threshold *p* = 0.001 voxel-level, cluster-level corrected using FWE *p* < 0.05.

⁎ Reduced threshold to *p* = 0.005 voxel-level, cluster-level corrected using FWE *p* = 0.05, as the clusters do not survive the FWE-cluster level *p* < 0.05 correction at *p* = 0.001 voxel-level.

Some regions were shared across different factors; specifically, the left angular gyrus was associated with phonological production, phonological recognition and semantic factors; the left posterior supramarginal gyrus and planum temporale were associated with phonological production and phonological recognition factors; the left anterior supramarginal gyrus was associated with phonological production and fluency factors; the posterior inferior and middle temporal gyri were associated with phonological recognition and semantic factors. Certain white matter tracts were also shared across different factors; specifically, the tract corresponding to the inferior longitudinal fasciculus was associated with phonological production and semantic factors; the anterior segment of the actuate fasciculus was associated with phonological production and fluency factors; and the posterior segment of the arcuate fasciculus was associated with phonological production, phonological recognition and semantic factors.

#### 3.2.4 Effect of lesion size

Given that some cortical regions are more likely to be damaged after MCA stroke than others (Phan et al., 2005), lesion volume was included (see Methods) as a covariate in subsequent VBCM analyses. When adding lesion volume to each of the verb and noun processing analyses (verb naming, noun naming, verb comprehension, and noun comprehension), even using a more liberal threshold (*p* = 0.005 voxel-level and *p* < 0.05 FWE-corrected cluster level), the verb naming and verb comprehension clusters dropped out of significance; whereas smaller clusters were identified for noun naming (cluster size = 1373 voxels) in the left angular gyrus, posterior supramarginal gyrus, and superior lateral occipital cortex; and for noun comprehension (cluster size = 1359 voxels) in the left inferior lateral occipital cortex, occipital fusiform cortex, temporal occipital fusiform cortex and posterior inferior temporal gyrus. Indeed, lesion volume correlated with behavioural performance on all four measures (verb naming: r = 0.65, verb picture-to-word matching: r = 0.67, noun naming: r = 0.62, and noun picture-to-word matching: r = 0.56, *p* < 0.0001).

In contrast, when adding lesion volume to the PCA-VBCM, the clusters remain significant. This outcome supports the proposal that PCA generates statically independent components, and improves power by extracting factors of interest and removing sources of noise in excluded factors (see Butler et al., 2014; Halai et al., 2017). Results are shown in Fig. 5b. Three of the five components did not significantly correlate with lesion volume [phonological production (r = 0.22, *p* = 0.12); phonological recognition (r = 0.18, *p* = 0.21); and executive function (r = 0.14, *p* = 0.33)], and therefore controlling for lesion volume did not have a significant effect on the neural correlates identified from the previous analysis conducted without accounting for lesion volume, as the clusters were smaller in size but they covered the same regions. The remaining two factors did correlate with lesion volume and thus partialling out this variable had some effect on the lesion correlates. Firstly, the neural correlates for the semantic factor (correlation with lesion volume: r = 0.37, *p* = 0.01) showed a reduction in cluster size and were limited to the left superior and inferior lateral occipital cortex, precuneus, and occipital fusiform gyrus. Secondly, there were no significant clusters remaining for the fluency factor (correlation with lesion volume r = 0.59, *p* < 0.0001).

## 4 Discussion

### 4.1 Behavioural profiles of noun and verb processing

A set of noun and verb materials was developed, matched simultaneously on word imageability, frequency, familiarity, age-of-acquisition, length and pictorial visual complexity. These materials were used to examine single-word noun and verb processing on both production and comprehension, across a large cohort of patients with post-stroke aphasia with a wide range of aphasia classification and severity levels. The findings revealed a lack of word class effect in both tasks once matched on several psycholinguistic features. Additionally, no association was found between noun versus verb performance and aphasia classifications. These results contradict previous case series and group studies showing noun-verb double dissociations in aphasia (e.g., Laiacona and Caramazza, 2004; Miceli et al., 1988; Zingeser and Berndt, 1990), or greater verb deficits compared to nouns among patients with post-stroke aphasia (e.g., Bastiaanse and Jonkers, 1998; Luzzatti et al., 2002; Mätzig et al., 2009). This discrepancy might reflect the fact that the materials used in this study were carefully matched on multiple semantic and psycholinguistic features simultaneously including imageability that (i) proved to be a factor that partially predicted naming and comprehension performance and (ii) tends to be substantially lower for most verbs than nouns. Nevertheless, these previous findings that showed greater verb production deficits compared to nouns were replicated in this study once unmatched noun and verb materials were used. This could explain the differences in single-word noun or verb processing that have been previously observed in single-case and case series studies, in which the deficits could reflect difficulties associated with either noun or verb items secondary to their psycholinguistic features.

The results from this study complement and add strong weight (given the large number and wide range of participants, matched stimulus sets, tackling both production and comprehension, and the use of PCA) to the semantic explanation of previously reported word class differences in single-word tasks (Bird et al., 2000; Luzzatti et al., 2002), and to previous findings showing that imageability and age-of-acquisition were strong predictors for naming performance in aphasia (e.g., Bird et al., 2000; Mätzig et al., 2009; Nickels and David, 1995). A range of factors in addition to semantic differences (such as morphological and syntactic factors that are language-dependent) may well play a key role in the noun-verb differences observed in sentence and narrative processing (Kemmerer, 2014; Vigliocco et al., 2011).

A data-reduction approach (PCA) was further utilised in this study to amalgamate noun and verb performance with detailed neuropsychological and aphasiological assessments. Again, this analysis revealed a lack of behavioural differences between noun and verb processing: the loading pattern for all noun and verb tests was entirely similar, with no additional extracted factor for verb processing only. More broadly, these findings are consistent with models proposing that language functions and different types of words are supported by a set of interacting general language components (Patterson and Lambon Ralph, 1999; Seidenberg and McClelland, 1989; Ueno et al., 2011; Ueno et al., 2014), rather than dedicated representations or processes for each language activity or word type (Caramazza and Hillis, 1991; Miceli et al., 1984).

### 4.2 Neural correlates of noun and verb processing

The lesion-symptom mapping showed that noun and verb processing (both production and comprehension) are jointly supported within wide cortical regions encompassing the left temporal and parietal lobes, and the underlying white matter tracts. These shared regions correspond to the ventral language pathway (Hickok and Poeppel, 2004; Parker et al., 2005; Saur et al., 2008; Ueno et al., 2011), and are consistent with previous studies showing that verb deficits are associated with lesions in left posterior temporal lobe and parietal regions, and that noun deficits can follow lesions to the left middle and inferior temporal gyri (Aggujaro et al., 2006; Hillis et al., 2002; Silveri et al., 2003). The current result are also consistent with functional neuroimaging studies on healthy participants showing a common and distributed neural representation for noun and verb processing, with activation across widespread cortical regions extending from occipital to temporo-parietal regions (Siri et al., 2008; Sörös et al., 2003; Tyler et al., 2001). The current findings aligns with the review, which indicated that nouns and verbs recruit a shared neural network and that no neural correlate differences between nouns and verbs emerge once semantic differences are properly controlled (Vigliocco et al., 2011). In the current study, noun and verb items were pairwise matched on several psycholinguistic and semantic features.

Additional frontal regions have been identified in the individual maps with verb but not noun processing, for both naming and comprehension. This is in line with other neuropsychological and neuroimaging studies (e.g., Daniele et al., 1994; Shapiro et al., 2006; Siri et al., 2008). The involvement of frontal regions with verb processing could reflect the activation of frontal motor regions that are associated with the motor planning of the actions to which the verbs refer, or the articulation of morphological markers (-*ing*) attached to the verbs during naming task. Furthermore, parietal regions along with white matter tracts corresponding to the anterior segment of the arcuate fasciculus have been identified as the only regions associated with verb comprehension over-and-above noun comprehension in the direct contrast. The importance of these parietal regions with verb processing has been shown in previous neuroimaging studies (Shapiro et al., 2006; Shapiro et al., 2005). It must be noted that lesion-symptom mapping performed on a single noun or verb tests should be treated with some caution, due to their high correlation with lesion volume.

In subsequent analyses investigating the neural correlates related to naming and comprehension revealed shared regions along the left temporal and parietal lobes and the underlying white matter tracts. Additional fronto-parietal regions, however, were involved with production but not comprehension. This is consistent with other findings showing the association of the same frontal areas with production in healthy controls (Saur et al., 2008), and aphasia (e.g., Lacey et al., 2017).

### 4.3 Neural correlates of principal language and cognitive components

The neural correlates associated with each of the five principal language and cognitive components (phonological production, phonological recognition, semantics, fluency and executive functions) derived from a combination of the patients' performance on the noun and verb tests as well as their background aphasiological and neuropsychological tests were also explored. Lesion correlations with these PCA factors benefit not only from the fact that these components are statistically independent but they also combine data and remove sources of noise. Indeed, the resultant lesion maps are very robust and also survive the inclusion of lesion volume. The results align with previous studies of each language and cognitive domain. The phonological production factor was uniquely associated with posterior parietal regions, that have been previously shown to be involved with speech repetition and phonological retrieval (Fridriksson et al., 2010; Hartwigsena et al., 2010; Kümmerer et al., 2013; Schwartz et al., 2012). This factor was also associated with the white matter tracts underlying inferior parietal regions, which correspond to the posterior segment of the arcuate fasciculus. This region corresponds to dorsal language pathway, and has been linked to phonological processing (Catani et al., 2005; Fridriksson et al., 2010; Parker et al., 2005; Saur et al., 2008). The phonological recognition factor was uniquely associated with Heschl's gyrus, which is critically involved with processing speech sounds (DeWitt and Rauschecker, 2012). Both phonological production and recognition factors were associated with tissues deep in the left planum temporale, which has been associated with speech recognition in previous studies (e.g., Mirman et al., 2015).

The neural correlates associated with the semantic factor were the largest in size and included anterior and posterior temporal regions, which have been linked to semantic processing, including semantic control in both patients, fMRI and TMS studies (Lambon Ralph et al., 2017; Noonan et al., 2013). Posterior left central perisylvian regions (angular gyrus, posterior inferior and middle temporal gyrus) were shared between the semantic, phonological production and phonological recognition factors. These posterior regions have been implicated in phonological and semantic processing by functional MRI studies, and the parietal regions have been associated with default mode network (cf. meta-analysis by Binder et al., 2009; Humphreys and Lambon Ralph, 2015; Noonan et al., 2013). The phonological production and semantic factors were also associated with white matter tracts that connect posterior occipito-temporal regions to the temporal pole, corresponding to the inferior longitudinal fasciculus. This underlying temporal stem corresponds to the ventral language pathway and has been shown to be involved with recognition and comprehension (Catani and Mesulam, 2008; Saur et al., 2008).

As well as various left pre-frontal and motor regions, the fluency factor was also associated with white matter tracts most likely corresponding to the frontal aslant tract that connects the medial superior portion of the frontal lobe to the inferior-lateral frontal region (Rojkova et al., 2015). These cortical regions and the aslant tract have been implicated previously with speech fluency in post-stroke aphasia (Halai et al., 2017) and in primary progressive aphasia (Catani et al., 2013). Finally, the executive function factor, which explained the least behavioural variance, did not uniquely correlate with any brain regions, unless the statistical threshold was reduced. At a lower threshold, this factor was associated with left frontal regions, which have been implicated with executive processing in both fMRI studies on healthy participants and in aphasia (Duncan and Owen, 2000; Lacey et al., 2017). It should be noted that these neural correlates were identified using univariate lesion-symptom mapping analyses and there have been suggestions that such approaches might bias the micro-level location of clusters (Mah et al., 2014). In the current study, the intention was not to discern the micro-level locations for verb and noun processing, but to establish that the differences between the two are not evident both behaviourally and neurally once multiple psycholinguistic variables have been accounted. That being said, future studies could further confirm findings from this study using multivariate approaches, although they too come with challenges such as determining a threshold on weights.

### 4.4 Conclusion

A novel noun and verb tests matched on several psycholinguistic features were developed (provided in Supplementary Appendix A). These neuropsychological tests probe production and comprehension, and can be used in neuropsychology and psycholinguistic research concerning word class effects. The tests can also be used in clinical practice for different populations with language impairments including aphasia, traumatic brain injury and neurodegenerative diseases. By matching important psycholinguistic variables, the tests offer the opportunity to obtain a better estimation of a patient's relative abilities on nouns and verbs. The verb tests can be used alongside existing (noun-based) clinical assessment tools, to provide a specific measure and baseline of verb retrieval and verb comprehension deficits, areas of challenge for most patients with language impairments. By using these novel tests on chronic post-stroke aphasia, no behavioural differences emerged between noun and verb processing at single-word level. In addition, noun and verb processing were found to share neuro-anatomical correlates within distributed cortical regions comprising the left temporal and parietal lobes, and their underlying white matter tracts. When noun and verb processing abilities were taken into the wider context of the aphasiological profile, it becomes apparent that existing principal components (phonological production, phonological recognition, semantics, fluency and executive functions) can account for these processes. The results overall provide evidence to models suggesting that language functions and different types of words are supported by a set of interacting general language components (Patterson and Lambon Ralph, 1999; Seidenberg and McClelland, 1989; Ueno et al., 2011; Ueno et al., 2014).

The following are the supplementary data related to this article.

Supplementary Appendix A List of noun and verb matched set along with the distractors constructing the noun and verb picture-to-word matching tests. Supplementary Appendix A

Supplementary Appendix B Participants' scores on background neuropsychological assessments. Supplementary Appendix B
