# Supplementary material for: Noun and verb processing in aphasia: Behavioural profiles and neural correlates
Source: Neuroimage Clin. 2018 Jan 31;18:215–30. doi: 10.1016/j.nicl.2018.01.023 (PMC5984597; doi:10.1016/j.nicl.2018.01.023)
Supplement: Supplementary Appendix A — List of noun and verb matched set along with the distractors constructing the noun and verb picture-to-word matching tests. [file mmc1.pdf]

Supplementary Appendix A

List of noun and verb matched set along with the distractors constructing the noun and verb picture-to-word matching tests

Target	Semantic distractor 1	Semantic distractor 2	Unrelated distractor 1	Unrelated distractor 2	Name agreement (picture naming)	Item accuracy (picture-to-word matching)
**Noun stimuli**						
**belt**	tie	leather	crack	sword	100	100
**bone**	skeleton	dog	tourist	belt	100	100
**brain**	idea	thought	witch	whistle	100	100
**brush**	comb	hair	rake	knot	88	100
**chain**	string	rope	fruit	stool	100	100
**collar**	shirt	tie	saddle	shower	100	100
**comb**	brush	hair	slide	office	100	100
**crack**	break	split	bone	chain	100	100
**cross**	church	star	king	picture	100	100
**fence**	wall	garden	road	root	100	100
**fruit**	vegetable	strawberry	shower	hammock	100	100
**hammock**	swing	beach	shadow	collar	100	100
**judge**	lawyer	court	belt	sword	100	100
**king**	queen	soldier	pocket	saddle	100	100
**knot**	chain	bow	stool	brain	100	100
**office**	library	reception	tourist	brush	100	100
**picture**	frame	wall	belt	fence	100	100
**pocket**	trousers	wallet	fence	road	100	100
**pond**	puddle	river	weight	comb	100	100
**rake**	hoover	broom	hammock	fruit	100	100
**road**	street	path	pocket	bone	100	100
**root**	trunk	leaves	brush	slide	100	96
**saddle**	seat	whip	whistle	judge	100	100
**shadow**	reflection	dark	pond	witch	100	100
**shower**	bath	water	picture	rake	100	100
**slide**	swing	slope	picture	brain	100	100
**stool**	chair	table	comb	crack	96	100
**sword**	dagger	knife	cross	weight	88	88
**tourist**	passenger	attractions	knot	shadow	88	100
**weight**	scale	tons	judge	pond	100	100
**whistle**	referee	mouth	office	king	100	100
**witch**	spells	wizard	collar	cross	100	100

Continued

Target	Semantic distractor 1	Semantic distractor 2	Unrelated distractor 1	Unrelated distractor 2	Name agreement (picture naming)	Item accuracy (picture-to-word matching)
**Verb stimuli**						
**bleeding**	dripping	flowing	washing	skating	96	100
**climbing**	falling	jumping	biting	ironing	100	100
**dancing**	holding	hugging	sleeping	washing	100	100
**diving**	swimming	sinking	running	biting	100	100
**drilling**	digging	puncturing	walking	shaving	100	100
**drinking**	eating	spitting	sewing	raking	100	100
**driving**	riding	cycling	tickling	stroking	100	100
**fishing**	shooting	catching	lighting	singing	100	100
**flying**	cruising	floating	drilling	knocking	100	100
**ironing**	hanging	drying	dropping	climbing	100	100
**juggling**	catching	balancing	yawning	sleeping	100	100
**jumping**	climbing	skipping	floating	shooting	100	100
**kicking**	scoring	throwing	rocking	pulling	100	100
**kissing**	licking	loving	flying	sewing	100	96
**knitting**	weaving	sewing	crossing	laughing	96	100
**laughing**	crying	smiling	ironing	knitting	100	100
**leaning**	bending	standing	writing	stirring	100	100
**marching**	striding	fighting	melting	pointing	100	100
**painting**	drawing	colouring	dancing	rocking	100	100
**raking**	sweeping	hoovering	drinking	praying	88	96
**reading**	writing	scanning	dripping	folding	100	100
**running**	walking	racing	juggling	cooking	96	100
**shooting**	fishing	hitting	barking	combing	100	100
**sitting**	crouching	lying	singing	planting	100	100
**skating**	skiing	sliding	catching	building	100	100
**skiing**	sliding	skating	cooking	waving	100	100
**smiling**	laughing	pouting	fishing	opening	100	100
**smoking**	lighting	coughing	marching	sliding	100	96
**snowing**	raining	hailing	peeling	riding	100	100
**swimming**	diving	snorkelling	waving	sitting	100	100
**walking**	marching	running	stroking	smiling	100	96
**writing**	typing	drawing	sneezing	bleeding	100	100

Please cite the paper when using the above tests. Thank you.
