# Supplementary material for: Noun and verb processing in aphasia: Behavioural profiles and neural correlates
Source: Neuroimage Clin. 2018 Jan 31;18:215–30. doi: 10.1016/j.nicl.2018.01.023 (PMC5984597; doi:10.1016/j.nicl.2018.01.023)
Supplement: Supplementary Appendix B — Participants' scores on background neuropsychological assessments. [file mmc2.pdf]

Supplementary Appendix B

Participants' scores on background neuropsychological assessments

ID	MP NW	MP W	Rep NW-I	Rep NW-D	Rep W-I	Rep W-D	WPM spoken	WPM written	CSB naming	BNT	CCT	SJ	VSJ	CAT	Ravens	Brixton	Digit-F	Digi-B	WPM	TRR	MLU	TOK
NH	98.61	98.61	86.67	80.00	100.00	100.00	98.44	98.44	93.75	88.33	84.38	86.46	86.25	90.63	94.44	70.91	62.50	57.14	32.81	90.91	52.64	6.98
Ebe	93.06	95.83	66.67	36.67	81.25	78.75	100	98.44	82.81	38.33	90.63	83.33	68.75	75.00	66.67	47.27	50.00	28.57	26.89	67.86	75.39	17.78
KA	95.83	95.83	83.33	70.00	96.25	96.25	100	100	84.38	61.67	84.38	79.17	80.00	78.13	77.78	65.45	87.50	42.86	43.68	72.97	66.22	23.49
PBL	79.17	80.56	0.00	0.00	38.75	23.75	100	100	34.38	15.00	92.19	89.58	81.25	71.88	97.22	80.00	37.50	0.00	12.97	55.26	16.06	12.06
DL	87.50	97.22	50.00	36.67	85.00	90.00	100	100	78.13	76.67	92.19	95.83	93.75	100.00	94.44	74.55	62.5	42.86	23.36	51.06	69.40	29.84
TK	88.89	81.94	0.00	3.33	35.00	22.50	100	100	15.63	8.33	84.38	71.88	75.00	87.50	69.44	54.55	37.50	42.86	20.78	60.61	48.39	20.95
MH	93.06	98.61	0.00	3.33	13.75	7.50	100	98.44	6.25	5.00	89.06	69.79	53.75	59.38	80.56	74.55	25.00	28.57	51.95	65.45	47.30	17.46
DCs	97.22	97.22	40.00	56.67	72.50	68.75	100	98.44	67.19	43.33	95.31	91.67	90.00	93.75	100.00	81.82	62.50	57.14	9.65	80.65	48.39	9.84
KS	94.44	95.83	73.33	80.00	93.75	95.00	71.88	67.19	31.25	13.33	68.75	84.38	66.25	84.38	86.11	52.73	100.00	57.14	45.90	75.31	62.26	25.71
RH	95.83	95.83	3.33	3.33	21.25	5.00	96.88	96.88	3.13	1.67	89.06	89.58	86.25	62.50	83.33	61.82	25.00	28.57	44.59	50.74	96.78	64.44
WE	87.50	91.67	46.67	30.00	71.25	67.50	100	100	78.13	55.00	87.50	87.50	87.50	84.38	91.67	70.91	62.5	42.86	26.07	59.42	58.94	21.90
Gha	86.11	93.06	80.00	46.67	91.25	98.75	95.31	96.88	87.50	78.33	92.19	95.83	93.75	93.75	83.33	69.09	50.00	42.86	22.51	55.17	85.32	36.83
DF	90.28	95.83	53.33	10.00	93.75	41.25	100	96.88	87.50	50.00	92.19	78.13	77.50	62.50	88.89	43.64	37.50	28.57	23.36	72.34	50.94	14.92
RL	56.94	59.72	13.33	16.67	61.25	53.75	96.88	98.44	84.38	63.33	95.31	93.75	98.75	62.50	80.56	72.73	62.50	57.14	28.33	77.50	42.45	12.70
JBr	90.28	97.22	90.00	80.00	97.50	95.00	100	100	93.75	85.00	93.75	93.75	93.80	96.88	97.22	67.27	75.00	71.43	46.51	70.11	87.44	27.62
Ebo	98.61	97.22	100	90.00	100	100	100	100	89.06	55.00	90.63	90.63	77.50	87.50	97.22	69.09	50.00	57.14	26.45	66.07	75.39	17.78
WC	79.17	76.39	30.00	10.00	76.25	71.25	98.44	98.44	54.69	55.00	75.00	94.79	83.75	75.00	52.78	70.91	62.50	42.86	46.09	56.56	86.59	38.73
BH	93.06	94.44	86.67	80.00	100.00	96.25	98.44	93.75	95.31	66.67	73.44	83.33	83.75	78.13	66.67	67.27	62.50	57.14	23.93	68.42	41.60	12.06
JS	75.00	93.06	50.00	46.67	90.00	88.75	100	100	89.06	43.33	95.31	96.88	97.50	81.25	100.00	65.45	50.00	57.14	50.14	46.98	100	100
AL	91.67	100	90.00	90.00	100	98.75	100	100	93.75	88.33	79.69	93.75	90.00	84.38	91.67	60.00	87.50	85.71	100.00	75.00	60.28	19.05
MB	98.61	98.61	70.00	66.67	87.50	72.50	100	100	87.50	73.33	90.63	87.50	87.50	93.75	97.22	76.36	50.00	28.57	20.92	89.58	73.86	15.24
DM	80.56	93.06	60.00	10.00	73.75	68.75	98.44	98.44	75.00	71.67	98.44	95.83	90.00	56.25	91.67	50.91	37.50	0.00	15.38	73.68	34.93	12.06
PW	73.61	84.72	0.00	0.00	0.00	0.00	100	95.31	4.69	5.00	78.13	73.96	67.50	56.25	50.00	34.55	25.00	0.00	7.24	39.13	26.60	14.60
JW	86.11	81.94	33.33	16.67	65.00	66.25	96.88	98.44	65.63	38.33	81.25	85.42	82.50	90.63	88.89	61.82	87.50	28.57	8.10	83.33	40.75	5.71
MAd	81.94	91.67	60.00	56.67	95.00	93.75	98.44	98.44	84.38	76.67	82.81	88.54	72.50	81.25	83.33	63.64	62.50	0.00	17.61	86.96	23.77	7.30
RR	88.89	86.11	10.00	3.33	51.25	42.50	98.44	100	54.69	23.33	92.19	82.29	80.00	56.25	88.89	43.64	25.00	28.57	13.94	67.74	34.81	9.84

Continued

ID	MP NW	MP W	Rep NW-I	Rep NW-D	Rep W-I	Rep W-D	WPM spoken	WPM written	CSB naming	BNT	CCT	SJ	VSJ	CAT	Ravens	Brixton	Digit- F	Digi-B	WPM	TRR	MLU	TOK
AD	95.83	93.06	23.33	13.33	57.50	55.00	96.88	98.44	53.13	50.00	85.94	82.29	73.75	68.75	63.89	30.91	75.00	42.86	12.01	60.00	20.37	7.94
KL	75.00	77.78	0.00	0.00	6.25	0.00	92.19	98.44	4.69	1.67	78.13	68.75	56.25	28.13	88.89	61.82	0.00	0.00	7.73	38.89	14.55	5.71
GP	98.61	95.83	43.33	46.67	95.00	95.00	98.44	100	71.88	56.67	93.75	89.58	86.25	78.13	97.22	78.18	37.50	28.57	26.63	52.13	60.56	29.84
JSc	75.00	86.11	36.67	63.33	90.00	91.25	98.44	98.44	71.88	53.33	82.81	76.04	72.50	75.00	77.78	43.64	62.50	42.86	15.56	78.57	42.02	8.89
AG	100.00	98.61	73.33	83.33	77.50	87.50	100	100	87.50	78.33	75.00	89.58	77.50	87.50	75.00	56.36	100.00	100.00	8.50	70.00	37.69	9.52
DC	91.67	97.22	0.00	0.00	0.00	0.00	100	98.44	4.69	0.00	82.81	78.13	65.00	75.00	88.89	76.36	37.50	42.86	49.84	47.95	96.78	46.35
CH	95.83	97.22	60.00	40.00	92.50	88.75	100	100	84.38	60.00	90.63	87.50	91.25	84.38	91.67	76.36	50.00	28.57	11.22	60.53	34.81	12.06
AS	55.56	52.78	0.00	0.00	36.25	0.00	76.56	59.38	6.25	1.67	60.94	42.71	38.75	50.00	47.22	47.27	25.00	0.00	8.50	33.33	10.19	1.90
MD	98.61	98.61	26.67	16.67	50.00	61.25	96.88	93.75	46.88	38.33	59.38	57.29	13.75	12.50	38.89	58.18	37.50	28.57	3.50	18.18	16.21	10.48
AB	80.56	87.50	26.67	13.33	86.25	63.75	95.31	98.44	76.56	41.67	79.69	75.00	57.50	75.00	88.89	88.89	37.50	28.57	26.59	51.64	84.33	38.73
PR	80.56	94.44	56.67	43.33	85.00	91.25	100	100	60.94	38.33	84.38	83.33	76.25	87.50	80.56	50.91	75.00	0.00	9.32	64.00	24.01	7.94
GL	98.61	97.22	93.33	63.33	100.00	81.25	96.88	95.31	68.75	31.67	73.44	75.00	60.00	65.63	91.67	58.18	37.50	28.57	6.01	48.57	20.61	22.22
GD	91.67	95.83	0.00	0.00	5.00	0.00	78.13	56.25	1.56	0.00	59.38	48.96	42.50	50.00	63.89	34.55	25.00	0.00	0.47	100.00	0.85	0.32
DB	87.50	58.33	70.00	30.00	85.00	83.75	64.06	76.56	7.81	8.33	82.81	59.38	41.25	31.25	86.11	40.00	37.50	28.57	11.33	33.33	44.94	38.10
JM	81.94	77.78	0.00	0.00	1.25	0.00	78.13	93.75	0.00	0.00	67.19	75.00	61.25	46.88	91.67	91.67	25.00	0.00	0.00	0.00	0.00	0.00
DR	97.22	86.11	53.33	20.00	88.75	38.75	62.50	92.19	14.06	3.33	65.63	46.88	57.50	46.88	83.33	65.45	37.50	0.00	15.50	29.31	26.32	18.41
Gho	47.22	43.06	16.67	3.33	62.50	32.50	85.94	60.94	25.00	16.67	53.13	45.83	32.50	43.75	61.11	34.55	25.00	0.00	3.54	66.67	24.20	3.81
JBo	95.83	93.06	23.33	6.67	43.75	35.00	100	96.88	39.06	10.00	73.44	65.63	76.25	34.38	77.78	60.00	75.00	42.86	2.43	90.91	7.13	3.49
PM	91.67	93.06	10.00	13.33	65.00	55.00	92.19	98.44	59.38	51.67	65.63	69.79	51.25	62.50	47.22	30.91	25.00	28.57	9.28	63.16	16.74	6.03
SL	84.72	86.11	0.00	0.00	0.00	0.00	59.38	65.63	0.00	0.00	62.50	52.08	48.75	34.38	88.89	52.73	0.00	0.00	0.00	0.00	0.00	0.00
CF	73.61	63.89	33.33	0.00	70.00	18.75	89.06	96.88	43.75	20.00	84.38	77.08	52.50	68.75	91.67	50.91	25.00	0.00	3.85	100.00	8.91	3.49
DBb	22.22	52.78	0.00	0.00	37.50	0.00	57.81	31.25	0.00	0.00	53.13	48.96	30.00	12.50	30.56	38.18	25.00	0.00	41.21	46.88	41.77	10.16

Scores are given as percentages.

Abbreviations: MP = minimal pairs, NW = non-words, W = words, Rep = repetitions, I =immediate, D = delay (Psycholinguistic Assessments of Language Processing in Aphasia (Kay, et al.,1992)), WPM = word-to-picture matching, CCT = Camel and Cactus Test, CSB = Cambridge Semantic Battery (Bozeat, et al., 2000), BNT = Boston Naming Test (Kaplan, et al., 1983), SJ = 96-trial synonym judgment test (Jefferies, et al., 2009), VSJ = verb synonym judgment test (Alyahya, et al., 2018), CAT = spoken sentence comprehension subtest from the Comprehensive Aphasia Test (Swinburn, et al., 2005), F = forward, B = backward digit span (Wechsler, 1987), WPM = words-per-minute, TOK = number of tokens, TTR = token-type ratio, MLU = mean length of utterance.
